# Supplementary material for: Association of Circulating miRNAs from the C19MC Cluster and IGF System with Macrosomia in Women with Gestational Diabetes Mellitus
Source: Int J Mol Sci. 2025 Aug 28;26(17):8367. doi: 10.3390/ijms26178367 (PMC12427975; doi:10.3390/ijms26178367)
Supplement: Supplementary file 1 [file ijms-26-08367-s001.zip › Figure S1 and Table S1.pdf]

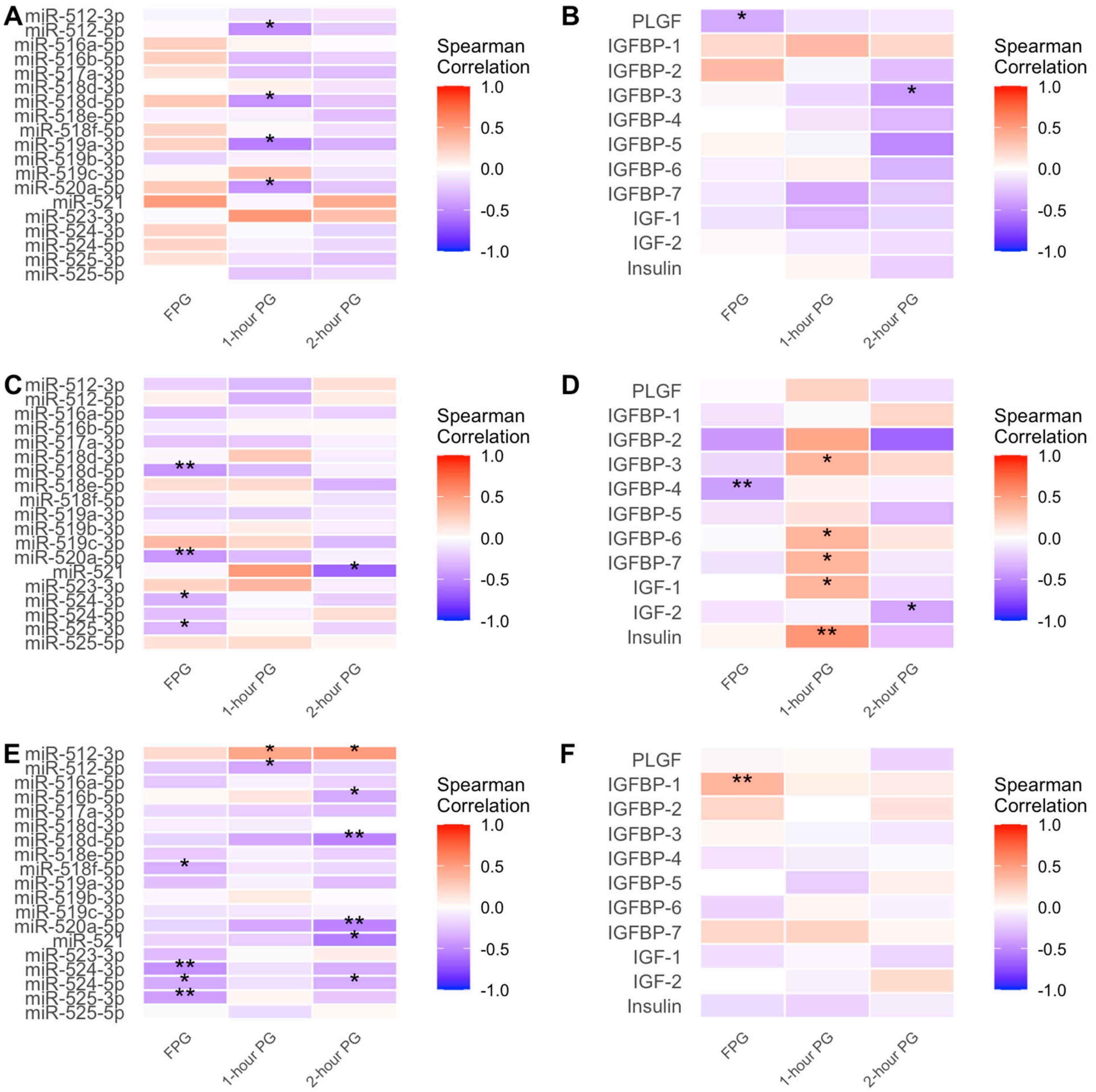

Supplementary Figure S1. Group-specific Spearman correlations between maternal glycemic values (OGTT) and (**A**,**C**,**E**) C19MC miRNA expression or (**B**,**D**,**F**) IGF-axis biomarkers and insulin concentrations. (**A**,**B**): normoglycemic pregnancies with appropriate-for-gestational-age (AGA) newborns; (**C**,**D**): GDM with AGA newborns; (**E**,**F**): GDM with large-for-gestational-age (LGA) newborns. Abbreviations: FPG = fasting plasma glucose; 1-hour and 2-hour = post-OGTT glucose; IGFBP = insulin-like growth factor-binding protein; PLGF = placental growth factor. ***** $p < 0.05$, ****** $p < 0.01$, ******* $p < 0.001$.

Table S1. Relative miRNA expression levels (2^-ΔCt) normalized to cel-miR-39 spike-in control in serum samples from the three study groups.

MicroRNA	Group 1 (n = 52)			Group 2 (n = 56)			Group 3 (n = 50)		
	Mean	Median	N Below detection	Mean	Median	N Below detection	Mean	Median	N Below detection
has-miR-512-3p	7.03×10^{-3}	1.29×10^{-3}	2	1.68×10^{-2}	7.55×10^{-4}	3	2.03×10^{-3}	5.28×10^{-4}	3
has-miR-512-5p	2.74×10^{-3}	4.56×10^{-4}	4	1.81×10^{-3}	5.14×10^{-4}	8	1.43×10^{-3}	5.23×10^{-4}	5
has-miR-516a-5p	5.01×10^{-4}	2.73×10^{-4}	3	2.73×10^{-2}	2.80×10^{-4}	2	4.04×10^{-4}	1.18×10^{-4}	2
has-miR-516b-5p	1.38×10^{-2}	4.83×10^{-5}	4	5.87×10^{-4}	1.18×10^{-4}	2	3.74×10^{-4}	1.15×10^{-4}	4
has-miR-517a-3p	4.27×10^{-3}	1.33×10^{-3}	7	3.37×10^{-3}	1.04×10^{-3}	9	2.51×10^{-3}	9.19×10^{-4}	6
has-miR-518d-3p	5.81×10^{-4}	5.88×10^{-5}	5	7.62×10^{-4}	5.24×10^{-5}	8	7.97×10^{-5}	3.28×10^{-5}	3
has-miR-518d-5p	1.80×10^{-5}	6.55×10^{-6}	3	1.17×10^{-5}	5.42×10^{-6}	8	1.50×10^{-5}	3.44×10^{-6}	4
has-miR-518e-5p	6.27×10^{-3}	1.67×10^{-4}	4	1.17×10^{-2}	2.52×10^{-4}	9	2.46×10^{-3}	1.46×10^{-4}	2
has-miR-518f-5p	5.99×10^{-5}	3.01×10^{-5}	3	2.21×10^{-2}	2.52×10^{-5}	8	6.47×10^{-5}	2.01×10^{-5}	4
has-miR-519a-3p	5.93×10^{-4}	5.71×10^{-5}	6	3.10×10^{-3}	2.50×10^{-5}	2	1.75×10^{-4}	6.39×10^{-5}	2
has-miR-519b-3p	8.95×10^{-3}	4.68×10^{-3}	5	1.73×10^{-2}	3.31×10^{-3}	2	3.03×10^{-3}	2.01×10^{-3}	4
has-miR-519c-3p	5.52×10^{-6}	1.64×10^{-6}	3	3.56×10^{-5}	1.03×10^{-6}	1	4.55×10^{-2}	1.68×10^{-6}	2
has-miR-520c-5p	1.80×10^{-5}	6.55×10^{-6}	4	1.17×10^{-5}	5.42×10^{-6}	6	1.50×10^{-5}	3.44×10^{-6}	4
has-miR-520g-3p	1.23×10^{-2}	9.28×10^{-3}	3	1.46×10^{-2}	1.26×10^{-2}	5	2.15×10^{-2}	1.59×10^{-2}	4
has-miR-521	1.57×10^{-4}	7.26×10^{-5}	6	7.71×10^{-4}	4.43×10^{-5}	3	7.11×10^{-5}	4.65×10^{-5}	5
has-miR-523-3p	1.50×10^{-6}	6.57×10^{-7}	3	1.54×10^{-2}	1.39×10^{-6}	3	5.00×10^{-2}	4.15×10^{-7}	3
has-miR-524-3p	1.46×10^{-2}	2.07×10^{-5}	3	8.96×10^{-3}	1.33×10^{-5}	3	9.07×10^{-3}	1.55×10^{-5}	4
has-miR-524-5p	6.63×10^{-3}	1.77×10^{-3}	6	4.15×10^{-3}	1.86×10^{-3}	6	3.67×10^{-3}	1.11×10^{-3}	4
has-miR-525-3p	1.37×10^{-4}	9.00×10^{-6}	5	8.38×10^{-3}	1.53×10^{-5}	5	6.38×10^{-5}	1.09×10^{-5}	4
has-miR-525-5p	2.16×10^{-4}	3.05×10^{-5}	7	1.54×10^{-4}	3.34×10^{-5}	7	6.85×10^{-5}	3.08×10^{-5}	5

Group 1 = normoglycemic pregnancies with normal birthweight newborns. Group 2 = gestational diabetes mellitus (GDM) with normal birthweight newborns. Group 3 = GDM with large-for-gestational-age (LGA) newborns.
